# Cross-cultural adaptation and validation of the STarT back screening tool in isiZulu

**DOI:** 10.4102/sajp.v76i1.1402

**Published:** 2020-06-01

**Authors:** Peta-Ann Schmidt, Vaneshveri Naidoo

**Affiliations:** 1 Department of Physiotherapy, Faculty of Health Sciences, University of the Witwatersrand, Johannesburg, South Africa

**Keywords:** STarT back screening tool, isiZulu, NSLBP, validity, reliability, translation

## Abstract

**Background:**

Non-specific low back pain (NSLBP) is one of the most prevalent conditions in the world. Identifying patients at risk for developing chronic NSLBP is key to effective treatment. The STarT back screening tool is a validated, prognostic screening tool identifying subgroups of NSLBP patients, and the risk factors associated with each subgroup, guiding treatment in the primary care of NSLBP.

**Objectives:**

To translate the English version of the STarT back screening tool into isiZulu and determine the content validity and reliability of the translated tool.

**Method:**

Translation was completed in four phases - forward translation and synthesis, backward translation and expert review. Validation included expert review for content validity and testing of the translated tool on 30 patients, determining test-retest reliability, internal consistency and usability.

**Results:**

Minor linguistic differences were addressed during the translation phase. Item content validity was excellent for relevance (1.00), satisfactory (0.94) for clarity, simplicity and ambiguity, with scale-content validity acceptable (0.955). Spearman’s correlation coefficient for test-retest reliability was acceptable (0.73). Cronbach’s alpha for internal consistency for the total score for test 1 and test 2 was 0.68 and 0.77, and for the psychosocial scale 0.62 and 0.77 respectively. Overall, 33% found the tool very easy to understand and 40% found it very easy to complete.

**Conclusion:**

The isiZulu STarT back screening tool showed excellent content validity, acceptable reliability and acceptable internal consistency.

**Clinical implications:**

Use of the isiZulu tool in local clinics and private practices can improve clinical decision-making and treatment outcomes for isiZulu-speaking patients with NSLBP.

## Introduction

Non-specific low back pain (NSLBP) is one of the most prevalent conditions in the world (Bruyere et al. 2012) and was the leading cause of disability worldwide in 2015 (Global Burden of Disease (GBD) Study 2015). The prevalence of NSLBP in African populations has been shown to be similar to the rest of the world (Louw et al. 2007). Many patients experience short episodes of acute NSLBP that resolve quickly, but others go on to develop chronic NSLBP, which can persist for up to 1 year after the initial onset (Hill et al. 2008). Approximately 23% of patients experience ongoing symptoms, of which 11% – 12% continue to experience long-term disability (Airaksinen et al. 2006). Chronic NSLBP thus places a significant socio-economic burden on society (Melloh et al. 2009), making diagnosis of patients at risk of developing chronic NSLBP very important.

Predicting the development of chronicity in NSLBP through the early identification of risk factors (Van Tulder et al. 2006) has been shown to lead to more effective treatment interventions (Melloh et al. 2009). Emphasis on psychosocial factors as risk factors for developing chronic NSLBP occurs in most low back pain clinical guidelines (Koes et al. 2010), and identification of the presence of such factors early in the course of NSLBP has been shown to improve outcomes of intervention (Hayden et al. 2010). Hill et al. (2008) developed and validated a back pain prognostic screening tool, the STarT (subgroups for targeted treatment) Back Screening Tool, which could be used to identify subgroups of patients with NSLBP and their associated risk factors (Keele University 2017), grouping these patients into low-, medium- and high-risk groups. A randomised controlled trial by Hill et al. (2011) compared a stratified care approach based on this back pain prognostic screening tool with current best practices and showed a significant improvement in patient outcomes and substantial economic benefits.

The STarT back screening tool is a self-administered questionnaire, and understanding the written language of this standardised tool is integral to its usage and interpretation. Understanding a language, written or spoken, involves the need to integrate new information with what is already available (Van Dyke 2016). Translation therefore gives different cultures the opportunity to access a huge amount of new information available in the English world, and in return allows the English world an understanding of other cultures. The STarT back screening tool was originally developed and validated in English and has been translated into 35 languages worldwide (Keele University 2017), and there is only one translated version of this tool available in any of the other official languages of South Africa, namely Afrikaans (The 11 languages of South Africa 2019).

Most South Africans, particularly in the African population, are bilingual and often use more than one language at home, with English usually considered a second or third language, and if a person is not able to use the language most familiar to them, they are unlikely to perform at their best, and their self-confidence will be undermined (The Importance of languages 2017).

Therefore, understanding the STarT back screening tool in their first language allows patients with NSLBP to use the tool more effectively, and it would assist practitioners to accurately group patients into low-, medium- and high-risk groups, which, in turn, would direct treatment interventions. Subsequently, validating the translated tool would determine how accurately it measures what it intends to measure. The objectives of this study were to cross-culturally adapt the STarT back screening tool into isiZulu and determine the content validity and reliability of the translated tool in an isiZulu population of NSLBP patients.

## Research methods and design

This study consisted of two phases – a cross-cultural adaptation phase, which included forward translation, translation synthesis, back translation and expert committee review, and a validity phase, which included testing of content validity, test–retest reliability, internal consistency and usability.

Participants of this study were purposively sampled on the basis of the guidelines established by Beaton et al. (2000) for the process of cross-cultural adaptation of self-reported measures. The participants in the cross-cultural adaptation phase of this study included two forward translators known to the researcher, two backward translators from Wits Translate (Wits Language Schools’ professional translation agency), and three healthcare professionals from the Department of Physiotherapy, who all made up the expert review committee.

Inclusion criteria for forward translation were a medically trained isiZulu first-language speaker or a university educated, non-medically trained isiZulu first-language speaker. For backward translation, services of two second-language English-speaking individuals, proficient in isiZulu and non-medically trained were utilised. For expert review, services of one healthcare professional proficient in isiZulu and English, two healthcare professionals proficient in English, with translation experience and the aforementioned forward and back translators were utilised.

The two forward translators translated the original English STarT back screening tool into isiZulu, developing translation 1 (T1) and translation 2 (T2). T1 and T2 were compared and discrepancies discussed until consensus on a common translation was reached. Synthesis of the two translated screening tools (T1 and T2) was conducted, and a new screening tool (T12) was produced. The two backward translators translated the T12 screening tool back into English, producing translations BT1 and BT2. The expert committee reviewed all the screening tools (T1, T2, T12, BT1 and BT2), discussed discrepancies and consolidated all the screening tools to develop a preliminary translated isiZulu STarT back screening tool.

The validation phase used a sample of convenience. For content validity, two forward translators, one backward translator and one healthcare professional from the expert committee were included. For test–retest reliability, internal consistency and usability, isiZulu South African men and women aged 18 years and older with acute or chronic NSLBP, with or without associated leg pain, willing to participate in this study, and able to return 2 weeks later for re-testing were included. They were recruited from Erasmus Physiotherapists Inc., Witbank General Hospital, Siphosensimbi Community Health Clinic and Lynnville Polyclinic (Mpumalanga Department of Health) in Witbank/eMalahleni. Non-university-educated, non-Zulu-speaking and reading persons were excluded when testing for content validity. Patients with potentially serious pathology (cauda equina, cognitive, neurological or rheumatologic disease, fracture of the spine and malignancy), current specific diagnoses such as disc herniation or spondylolisthesis, spinal surgery in the last 6 months, pregnancy, and those who do not read or speak isiZulu were excluded when testing test–retest reliability, internal consistency and usability.

Polit and Beck (2006) and Yaghmaie (2003) recommended a panel of four experts as the minimum number of participants required to determine item content validity (I-CVI) and scale-content validity (S-CVI). The minimum total sample size to determine test–retest reliability, internal consistency and usability was calculated to be 27 patients based on an intra-class correlation coefficient (ICC) of 0.9 and a maximum width of 0.23 (*N* = [16*p* (1-*π*)]/*w*^2^, where *p* is the expected ICC of 0.9 and *w* is the width of 0.23). The expected ICC of 0.9 and the maximum width of 0.23 were based on translation and validation studies conducted in Xhosa, Afrikaans and English populations by Morris et al. (2012).

The preliminary translated isiZulu STarT back screening tool was reviewed for I-CVI by the four experts and then tested on the intended patient population as recommended by Beaton et al. (2000).

Two weeks later a re-test of the preliminary translated isiZulu version on the same patient population was performed as conducted by Hill et al. (2008) in the validation of the English STarT back screening tool. Transport costs for each participant were reimbursed by the first author. A post-retest questionnaire was administered at this point to determine the feasibility (utility, efficiency, learnability and satisfaction [Nielsen 2012]) of the isiZulu STarT back screening tool (Glover & Albers 2007), where each patient reported on the ease of comprehension of the translated tool, the ease of completing the tool, the time needed to complete the tool (5, 10, 15 and 20 min) and any suggestions to improve the clarity of the tool. The participants were sent reminders via SMS to attend follow-up visits at the same place where they were recruited. There was no control for confounding factors during the 2 weeks until follow-up. After all testing and re-testing were completed, the pre-final version of the translated isiZulu STarT back screening tool was submitted to the original developers of the tool for approval of study methodology and outcome. Once the original developers of the STarT back screening tool were satisfied with the translation and validation process, the translated isiZulu tool was made available on the following website: https://startback.hfac.keele.ac.uk/training/resources/start-back-translations/.

The content validity was determined by calculating I-CVI and S-CVI, measuring the representativeness and comprehensiveness of the items in a scale (Yaghmaie 2003) as well as the degree to which the instrument measures a specific construct (Bolarinwa 2015). A four-point ordinal scale was used to rate each question/item based on relevance, clarity, simplicity and ambiguity (Yaghmaie 2003), which included the following: (1) not relevant (not clear/not simple/doubtful); (2) question needs some revision; (3) relevant (clear/simple/no doubt but needs minor revision); and (4) very relevant (clear/simple/meaning is clear). The item content validity was calculated as the number of experts giving a rating of 3 or 4 divided by the total number of experts for each question/item. The item content validity should be 1.00 when there are five or fewer experts (Polit & Beck 2006). This was carried out to ensure that the translated version of the STarT back screening tool was understood in the local context and language. Scale-content validity was subsequently calculated on the basis of these results by adding the I-CVI for each aspect (relevance, clarity, simplicity and ambiguity) and dividing by the total number of aspects to arrive at the average (I-CVI for relevance + I-CVI for clarity + I-CVI for simplicity + I-CVI for ambiguity/4). The S-CVI is the proportion of the total number of items that are content-valid, or the proportion of items that received a score of 3 or 4 by the experts (Polit & Beck 2006), and is thus the average number of items rated as 3 or 4 across the questions/items for relevance, clarity, simplicity and ambiguity (Polit & Beck 2006). The S-CVI of 0.80 or higher is considered acceptable (Polit & Beck 2006).

The test–retest reliability was measured using Spearman’s correlation coefficient. Correlation coefficients higher than 0.8 demonstrate excellent reliability, coefficients between 0.6 and 0.8 demonstrate good reliability, and coefficients below 0.6 show poor reliability. The Internal Consistency Index of reliability/homogeneity is the degree to which the questions or items in the tool measure the same thing (Bolarinwa 2015). The higher the Internal Consistency Index or reliability value, the more reliable the measure. The Internal Consistency Index of this tool was evaluated by calculating Cronbach’s alpha coefficients for the total score (questions 1–9) and for the psychosocial subscale (questions 5, 6, 7, 8 and 9). An alpha index equal to or more than 0.90 is considered excellent (Bolarinwa 2015), equal to or more than 0.80 is considered good, equal to or more than 0.70 is considered acceptable, equal to or more than 0.60 is considered questionable, whilst less than 0.60 shows poor internal consistency.

Descriptive statistics analysed the data regarding socio-demographic information and previous history of low back pain and the information pertaining to the usability of the tool. Information about dropouts and missing data was also recorded.

### Ethical consideration

Ethical approval to conduct the study was obtained from the Health Research Ethics Committee (HREC) of the University of the Witwatersrand (Ethical Clearance Number: M170717), and permission to translate and validate the STarT back screening tool into isiZulu was obtained from the original authors at the Keele University in North Staffordshire, UK.

## Results

### Phase 1: Translation

Minor linguistic differences emerged during the forward and back translation phases, which were addressed in consultation with the expert committee. The word ‘sometime’ in questions 1 and 2 of the English tool was translated to mean ‘other time’ in isiZulu, with no word meaning exactly the same in isiZulu. The words ‘more slowly’ in question 4 were translated to mean ‘long time’ in isiZulu, as again no isiZulu word was found for ‘slowly’. In question 5, the words ‘physically active’ were initially translated to mean ‘mostly exercise’ with a bit of other activity, but the expert committee agreed that it relates to being physically active during activities of daily living (ADLs) and not specific exercises. The translation was thus changed to mean ‘general activity’, which better aligns with the original text. The translators agreed that the term ‘worrying thoughts’ in question 6 could be translated to mean worry or stressful. It was decided that *engikhatazayo* which means ‘bothering me’ was the closest translation, as worry and stressful are understood as the same concept in isiZulu. The forward and back translators found the word ‘terrible’ in question 7 difficult to translate as no specific word exists in isiZulu. The expert committee agreed to *ngizwasengathi*, which means ‘I feel like’ and better covers the original meaning of the word. Difficulty was found in translating ‘in general’ and ‘enjoy’ in question 8, as no direct translated words exist in isiZulu. The expert committee revised the full sentence to convey the meaning of both these terms – *Angizange ngizithokozele izinto engivame ukuzijabulisa ngazo njengenjwayelo*, which translates as ‘I did not enjoy the things I usually enjoy’. The word ‘bothersome’ in question 9 is not a word often used in Africa, and does not exist in isiZulu, so the expert committee agreed that the most common translation *kukuhluphe kangakanani* will be used, meaning ‘to bother or trouble’.

The above changes were made, and the preliminary final translation was resubmitted to the expert committee for editing. No further changes were required, and this preliminary final translation was used in the validation process.

### Phase 2: Validation

A summary of the demographic characteristics of the sample population is given in Table 1. A total of 37 patients was recruited to participate in our study, with seven patients not returning for the second round of testing. Two questionnaires were declared invalid because of missing data. Overall, 76% of the sample population consisted of women with 40% aged between 50 and 59 years. Educationally, 53% were high school educated, 60% were unemployed, and 50% had no previous history of back pain. Finally, 60% of the sample population was allocated to the high-risk group after tests 1 and 2.

**TABLE 1:** Demographic characteristics (*n* = 30).

Variable	Category	Frequency (*n*)	%
Gender	Male	7	23
	Female	23	76
Age	21–29 years	4	13
	30–39 years	2	6
	40–49 years	8	26
	50–59 years	12	40
	60 years and older	4	13
Level of education	Primary school	10	33
	High school	16	53
	University	4	13
Occupation	Sedentary	3	10
	Non-sedentary	9	30
	Unemployed	18	60
Hours worked	40 h per week	7	23
	Less than 40 h per week	4	13
	Retired	3	10
Previous history of back pain	None	15	50
	Falls	5	16
	Physical violence	1	3
	Motor vehicle accidents (MVAs)	2	6
	Back and neck injury	2	6
Risk groups Test 1	Low risk	6	20
	Medium risk	6	20
	High risk	18	60
Test 2	Low risk	6	20
	Medium risk	4	13
	High risk	20	60

### Content validity

The content validity was measured in two components, I-CVI and S-CVI. The item content validity for relevance was excellent (means I-CVI = 1.00) and was satisfactory for clarity, simplicity and ambiguity (means I-CVI = 0.94). The S-CVI was calculated by adding the I-CVI for each category (relevance, clarity, simplicity and ambiguity) and dividing by the total number of categories to reach the average. The S-CVI for the final translated isiZulu STarT back screening tool was 0.955, agreeing with Polit and Beck’s (2006) recommendation of an S-CVI being considered acceptable with a value of 0.80 or higher.

### Test–retest reliability

The test–retest reliability was determined by calculating Spearman’s correlation coefficient (ρ) between test 1 and test 2, resulting in ρ = 0.73, which demonstrates *acceptable* test–retest reliability (Glen 2018) (Table 2).

**TABLE 2:** Test–retest reliability.

Sample size (*n*)	Spearman’s correlation coefficient (ρ)	Significance level (*p*)	95% confidence interval (95% CI)
30	0.73	0.0001	0.381–0.820

### Internal consistency

The internal consistency was evaluated by calculating Cronbach’s alpha coefficients (item by item) for questions 1–9. An alpha index of more than 0.70 is considered to be acceptable internal consistency (Bolarinwa 2015).

Cronbach’s alpha was calculated for the total score (questions 1–9) as 0.68, which indicates less than acceptable internal consistency, and for the psychosocial subscale (questions 5, 6, 7, 8 and 9) as 0.68, which also showed less than acceptable internal consistency.

### Usability

**FIGURE 1:**
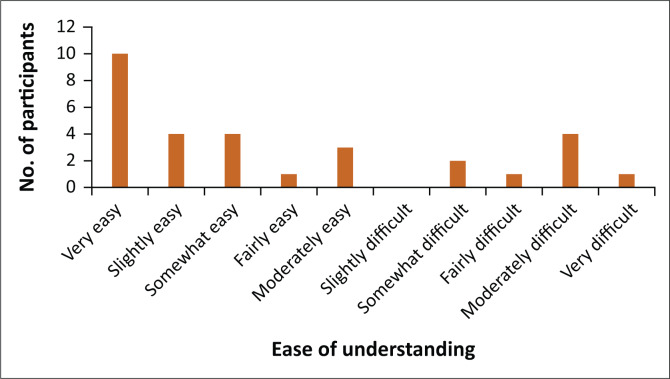
Ease of understanding the translated tool (*n* = 30).

Figure 1 demonstrates usability of the developed questionnaire: 33% found the translated tool very easy to understand, whilst 3% found it very difficult to understand.

**FIGURE 2:**
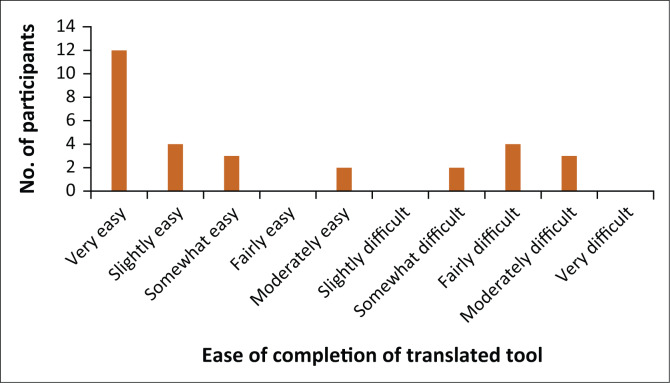
Ease of completion of the translated tool (*n* = 30).

Figure 2 demonstrates the ease of completion of the developed questionnaire: 40% found the translated tool very easy to complete, whilst 10% found it moderately difficult to complete.

**FIGURE 3:**
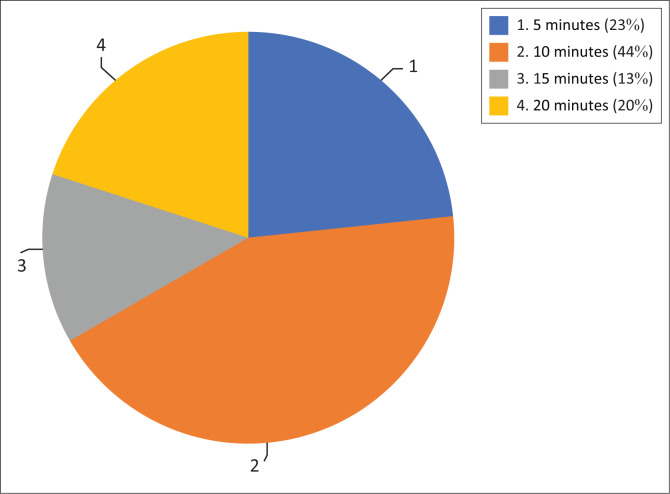
Time to complete the translated tool (*n* = 30).

Figure 3 demonstrates the time taken to complete the developed questionnaire: 20% took 20 min to complete the translated isiZulu tool, 13% completed it in 15 min, 44% took 10 min and 23% completed it in 5 min.

Table 3 demonstrates suggestions from the participants: 27% of the participants requested assistance with the explanation of the questions.

**TABLE 3:** Participants’ suggestions (*n* = 30).

Suggestions	Frequency (*n*)	Percentage (%)
None	19	63
Include more questions about lifting, bending and standing	1	3
Prefer tool in English	1	3
Need assistance with explanation of questions	8	27
Title of tool needs to be translated	1	3

## Discussion

### Phase 1: Translation

**FIGURE 4:**
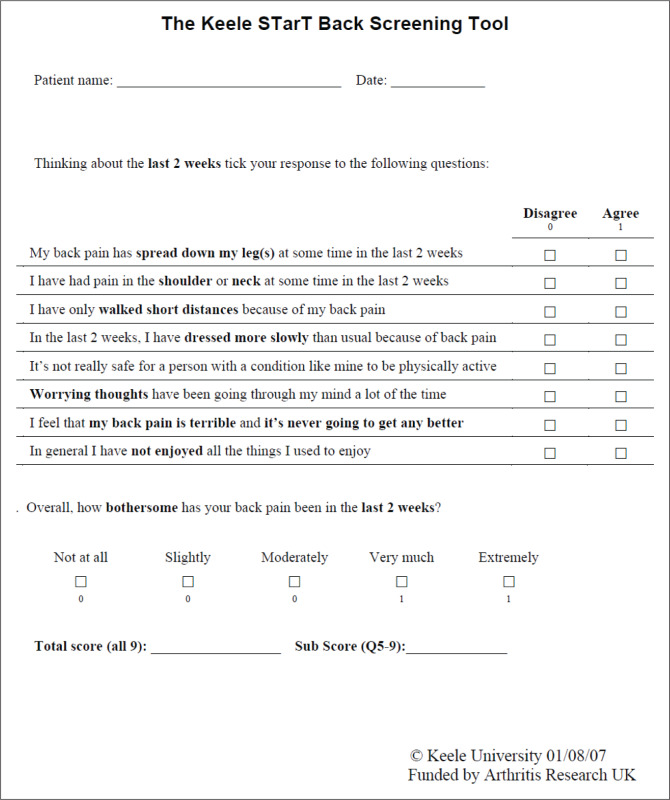
English STarT back screening tool.

**FIGURE 5:**
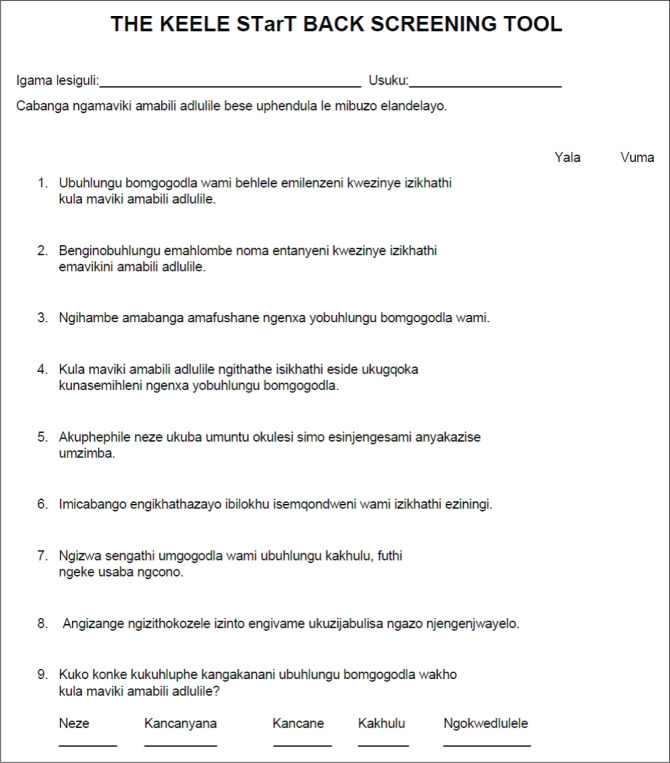
isiZulu STarT back screening tool.

The translation procedure applied in this study followed the guidelines set out by Beaton et al. (2000). The English STarT back screening tool and the final isiZulu STarT back screening tool are provided in Figures 4 and 5, respectively.

Finding equivalent words without altering the meaning of the original sentence was the major problem experienced during the translation phase and was resolved through discussions within the expert committee.

Firstly, the closest possible translation for a specific word was used; secondly, the closest possible translated meaning for a phrase was used; and thirdly, sentence structure was adjusted to convey the closest possible meaning to the English version. Some words had no equivalent in isiZulu and were translated to the closest possible meaning, such as ‘sometimes’ in questions 1 and 2, ‘worrying’ in question 6 (similar difficulty was faced in the French study; Bruyere et al. 2012), ‘terrible’ in question 7 (similar difficulty was faced in the Finnish study; Piironen et al. 2016), ‘enjoy’ in question 8 and ‘bothersome’ in question 9 (similar difficulty was faced in the Finnish, Piironen et al. 2016, and the Danish, Morso et al. 2011 studies). Certain phrases such as ‘physically active’ in question 5 posed difficulties for both forward and back translators, as well as ‘in general’ in question 8. Similar difficulties were experienced in the German (Aebischer et al. 2015), Swedish (Betten et al. 2015), Italian (Maggiani & Abenavoli 2019) and Finnish (Piironen et al. 2016) translation studies. Ultimately the expert committees in all these studies decided to keep the wording as close to the original English version as possible so as not to lose the meaning entirely. All translation challenges and changes to the isiZulu version were addressed and approved by the expert committee before testing the validity, reliability and usability of the translated tool.

### Phase 2: Validation

South Africa has one of the most diverse populations on the African continent, with 11 official recognised languages. Almost one-quarter (23%) of the population speaks isiZulu as their home language, and 58% speak it as their second language. English is the dominant language in major cities across South Africa but is only spoken as a second language by black South Africans in most other and rural areas of the country, including Witbank/eMalahleni, Mpumalanga, where up to 24% speak isiZulu (The 11 languages of South Africa 2019).

The majority of the sample population consisted of women (77%) aged between 50 and 59 years, which is similar to the literature showing a higher prevalence of low back pain onset in women aged between 30 and 50 years (Nicholas et al. 2011; Yoshimoto et al. 2017), and which does not differ in the developed world versus the developing world (Louw et al. 2007). A number of other translation studies identified employment status of their participants (Hill et al. 2008; Karstens et al. 2015; Piironen et al. 2016; Robinson et al. 2017), highlighting that the majority of patients were employed or received disability benefits, whereas most participants (60%) in our study were unemployed and did not receive employment or disability benefits.

This is an expected difference, as our study is one of the only studies carried out in developing Africa, where resources are limited, income inequality exists and the majority of the population does not have access to disability benefits or unemployment funds (United Nations Development Program (UNDP) 2017).

Most of the sample population was high school educated (53%), which was similar to the Swedish (64%) (Betten et al. 2015), Portuguese (32%) (Raimundo et al. 2017) and Brazilian–Portuguese (60%) (Pilz et al. 2014) studies. None of the other translation studies, including the original English study, examined the educational levels of their sample populations, perhaps suggesting that educational levels would not influence their translation process. Educational levels may not be a concern in developed countries; however, in the developing world where disparity in education exists, level of education/literacy could impact on completing the questionnaire (United Kingdom [UK] Essays 2016). The legacy of apartheid presents a significant challenge for education and health systems in South Africa. Black South Africans people were on the receiving end of education inequality, resulting in lower educational levels, a lack of educational resources and a shortage of qualified teachers (UK Essays 2016). This has led to a lack of basic information regarding health and wellness, particularly amongst those above the age of 50 years (UK Essays 2016). This is demonstrated by the majority of this study’s participants (77% in the 50–59-year age category), and the age prevalence of NSLBP (30–60 years) (Maher, Underwood & Buchbinder 2017). Lower levels of education demonstrated in our study suggest that the participants do not have access to basic healthcare information as a result of education inequalities and healthcare challenges.

Most participants were stratified into the high-risk group (60%), which may suggest that the patients are not actively seeking treatment for their back pain, were not getting the correct treatment when their back pain began, or were unaware of the back care treatment options available. The possibility also exists that the staff at local primary care clinics in Mpumalanga was not educated on the correct treatment pathways for these patients, and thus did not know that these patients should be referred for physiotherapy or for interdisciplinary management.

Measuring a questionnaire’s content validity measures the questionnaire’s quality (Bolarinwa 2015), which is a necessary part of developing an appropriate tool. Acceptable content validity shows that the content is relevant and appropriate, particularly when adapting a specific tool culturally, as seen in our study. The Dutch translation study (Bier et al. 2017) and the original English tool (Hill et al. 2008) tested content validity based on floor and ceiling effects, contrary to our study. For floor and ceiling effects to be present, some items need to be missing in the lower and upper ends of the scale, which results in limited content validity (Terwee et al. 2007). Floor and ceiling effects were not tested in our study, as the recommended sample size (Terwee et al. 2007) to test these effects is at least 50 patients. This study comprised only 30 patients, and the sample size calculation in our study was primarily based on a similar translation study performed by Morris et al. (2012).

Other translation studies (Morris et al. 2012) tested face and content validity simultaneously, using a group of patients to report the ease of understanding and ease of completing the translation. Our study did not test face validity, as it is an even more subjective test than content validity (Bolarinwa 2015), but used a panel of experts to test content validity, and the sample population to report on usability of the isiZulu tool. Criterion-related (predictive and concurrent) validity and construct validity were tested in other translation studies (Mphahlele, Mitchell & Kamerman 2008, Sharafi et al. 2017; Tsang Chi Chung et al. 2017), but were not tested in our study because the STarT back screening tool is a screening tool and does not predict future change. Furthermore, there is a lack of single reference isiZulu translated standard tools that screens for similar constructs.

The test–retest reliability of the preliminary isiZulu STarT back screening tool showed acceptable reliability (ICC of 0.73; 95% confidence interval [CI]: 0.38–0.82) for the total score, which is similar to that of the original version for the total score (ICC of 0.73; 95% CI: 0.57–0.84) (Hill et al. 2008). The Brazilian–Portuguese (Pilz et al. 2014), Portuguese (Raimundo et al. 2017), Finnish (Piironen et al. 2016) and German (Karstens et al. 2015) studies also demonstrated acceptable reliability (ICC ranging from 0.70 to 0.78).

Internal consistency in our study showed less than acceptable results for both the total score (0.68) and the psychosocial subscale (0.68). This was less than the original English version (0.74 total score and 0.74 psychosocial subscale) (Hill et al. 2008) as well as the Brazilian–Portuguese (Pilz et al. 2000), Portuguese (Raimundo et al. 2017), Finnish (Piironen et al. 2016), French (Bruyere et al. 2012), Persian (Abedi et al. 2015) and Japanese (Matsudaira et al. 2016) versions. Difference in the results of our study could be because of the way the constructs of the original English version were translated, with many words and phrases not existing in the isiZulu language. It may also be because of the level of education of our study sample not understanding the translated concepts clearly, particularly the negative wording of some of the questions (Bolarinwa 2015). Less than acceptable internal consistency resulted in less than acceptable reliability of the tool, which suggests the need to retest the translated tool on a different sample population.

Usability, tested in 30 participants, was found to be acceptable, easy to understand and easy to complete, whereas all the other translated versions, including the original English version (Hill et al. 2008), used a smaller sample size (*n* = 12–30), and also found good acceptability, ease of understanding and ease of completion (Abedi et al. 2015; Aebischer et al. 2015; Betten et al. 2015; Bier et al. 2017; Bruyere et al. 2012; Gusi et al. 2011; Hill et al. 2008; Karstens et al. 2015; Luan et al. 2014; Maggiani & Abenavoli 2019; Morso et al. 2011, Piironen et al. 2016; Pilz et al. 2000; Raimundo et al. 2017). The participants in our study took longer to complete the isiZulu questionnaire than the other aforementioned translation studies, which may be because of educational levels noted in the sample population, or because of this being the first time this sample population has been exposed to any form of health questionnaire.

The strengths of our study included the use of internationally recognised guidelines, the use of first-language isiZulu speakers throughout the process, which strengthened the linguistic accuracy of the translation, as well as the use of a wide inclusion criterion for the validation phase, and patient recruitment from a variety of geographical outpatient clinics which contributed to the acceptable external validity and reliability of our study. The limitations of our study included its inability to measure criterion or construct validity because of the lack of a single reference standard isiZulu translated tool against which the isiZulu STarT back screening tool could be tested. A smaller sample size than recommended by Terwee et al. (2007) could also have limited the validation results. The isiZulu tool also did not account for the treatment effect that NSLBP patients may have received between the first and second test–retest reliability testing, which could affect the outcome of the test–retest reliability test. However, the original STarT back screening tool was not designed to gather this type of information. The isiZulu tool was also only tested in the Mpumalanga province of South Africa, where many but not the majority of isiZulu-speaking people reside.

It is necessary to conduct another study to evaluate the psychometric properties in a larger, representative sample. Translation and validation of the Orebro Musculoskeletal Pain Screening Questionnaire (OMPSQ) into isiZulu, which then could be used as a reference standard to test the isiZulu STarT back screening tool could assist in fully validating the tool. It is also necessary to test the clinical effectiveness and cost-effectiveness of the isiZulu translation as well as the original English version in a South African context by comparing this stratified approach with a non-stratified best practice approach. This would determine whether stratified care leads to a significant decrease in healthcare costs and faster recovery periods specifically in South Africa.

isiZulu is the most widely spoken language in South Africa, so the widespread use of the isiZulu STarT back screening tool could have a significant effect on the way NSLBP is treated. Therefore, making the isiZulu tool available for use by clinicians in local clinics and private practices would improve clinical decision-making and treatment outcomes for isiZulu-speaking patients with NSLBP, and possibly improve physiotherapy and inter-professional referrals as needed. Training in ‘psychologically informed physiotherapy’ is also needed for treatment of the high-risk group.

## Conclusion

Established guidelines for the translation process were followed, and a preliminary isiZulu STarT back screening tool was produced, which showed good linguistic accuracy. This preliminary isiZulu tool was tested on a sample population of isiZulu-speaking patients presenting with NSLBP, and they reported good understanding and ease of completing the tool. The translated tool showed good content validity, acceptable test–retest reliability and less than acceptable internal consistency. Therefore, the preliminary isiZulu STarT back screening tool could be used in an isiZulu population presenting with NSLBP.
